# Caution for Diagnosis and Surgical Treatment of Recurrent Cholangitis: Lessons From 5 Cases of Bile Duct Tumor Thrombus Without a Detectable Intrahepatic Tumor

**DOI:** 10.1097/MD.0000000000000080

**Published:** 2014-08-29

**Authors:** Zheng Wu, Kun Guo, Hao Sun, Liang Yu, Yi Lv, Bo Wang

**Affiliations:** Department of Hepatobiliary Surgery (ZW, KG, HS, LY, YL, BW), First Affiliated Hospital of Xi’an Jiaotong University, Xi’an, Shaanxi, People’s Republic of China.

## Abstract

The hepatocellular carcinoma (HCC) patients with bile duct tumor thrombus (BDTT) usually have no specific clinical symptoms at early stages. HCC with BDTT was usually misdiagnosed when the intrahepatic tumor was small, even undetectable.

In this study, 5 cases of HCC with BDTT misdiagnosed as choledocholithiasis and cholangitis in the local hospital are described. We analyzed retrospectively and summarized our experiences of these 5 HCC patients with BDTT misdiagnosed in the local hospital during the past 5 years. The diagnosis, treatment, and outcome of the patients are discussed.

Three patients underwent hepatectomy with thrombectomy and T-tube drainage. One patient underwent hepatectomy with the resection of the common bile duct and hepatojejunostomy, and palliative surgery was performed in 1 patient with portal vein tumor thrombus and intrahepatic metastasis. The patients were followed for 6–22 months; 4 patients died of tumor recurrence and metastasis or hepatic failure, despite 3 of these patients having received transhepatic arterial chemotherapy and embolization or radiofrequency ablation therapy.

Early and accurate diagnosis of HCC with BDTT is very important. When patients have a history of abnormal recurrent cholangitis, HCC with BDTT should be highly suspected. Intraductal ultrasonography (US), intraoperative US, and histopathological examination are very valuable for the diagnosis. The prognosis of HCC patients with BDTT is dismal. Identification of this type of patient is clinically important, because surgical treatment may be beneficial.

## INTRODUCTION

Hepatocellular carcinoma (HCC) is one of the most common malignant neoplasms worldwide and is characterized by a low rate of early diagnosis and high mortality, especially in Eastern Asia. It is the fifth most common malignancy in men and the ninth most common malignancy in women.^1^ Worldwide, HCC contributes to more than 695,900 deaths annually.^2^ HCC generally spreads through the liver via the portal vein. Portal vein tumor thrombus is frequently observed in resected liver specimens and its incidence is high.^3,4^ Tumor thrombus is also detected within the bile duct, where they can cause obstructive jaundice. HCC with bile duct tumor thrombus (BDTT) is relatively rare; its incidence is approximately 1.2%–9.0%, and its clinical and pathological characteristics remain to be defined.^5^

Jaundice in HCC patients is divided into hepatocellular and icteric types in terms of its underlying pathophysiology.^6^ Hepatocellular-type jaundice in patients with HCC is typically associated with advanced liver cirrhosis or extensive tumor infiltration to liver parenchyma that leads to hepatic insufficiency.^3,7^ For these patients, life expectancy is short, and aggressive treatment modalities, including surgery, are not recommended. Icteric-type jaundice is caused by obstruction of the bile duct by BDTT. Once BDTT in HCC patients extends to the common hepatic duct or common bile duct, it causes obstructive jaundice. The HCC patients with BDTT usually have no specific clinical symptoms or signs at early stages although modern diagnostic modalities are available.^8,9^ It is usually difficult to make accurate diagnosis before operation, because of low incidence and limited awareness of its clinical and imaging features to find the BDTT preoperatively.^10^ Because of insufficient knowledge on this disease, it tends to misdiagnose BDTT as choledocholithiasis and cholangitis, especially that intrahepatic tumor might be small, even undetectable.

In the present study, we analyzed retrospectively and summarized our experience of 5 HCC patients with BDTT misdiagnosed as choledocholithiasis and cholangitis in the local hospital during the past 5 years. To our knowledge, this is the first report describing cholangitis as the initial symptom of HCC.

## PATIENTS AND METHODS

### Patients

Five patients misdiagnosed as choledocholithiasis and cholangitis in the local hospital were confirmed HCC with BDTT by surgery and histology between 2007 and 2012 at our hospital. Five patients were admitted to the local hospital with initial symptoms (high fever, jaundice, and abdominal pain) of cholangitis. All the patients received emergency treatment after abdominal ultrasound examination and/or abdominal computed tomography (CT) scan. Three patients received choledocholithotomy and T-tube drainage therapy. The other 2 patients received emergency endoscopic nasobiliary drainage (ENBD) drainage; 1 case was confirmed as BDTT after thrombus extraction during endoscopic retrograde cholangiopancreatography (ERCP) and then transferred to our hospital for further treatment. The other patient received plastic stent placement after ENBD drainage, and further received choledocholithotomy and choledochojejunostomy because of recurrent cholangitis.

### Methods

Laboratory data about the patients before surgery were recorded and analyzed. The patients received 2 or more preoperative diagnostic imaging procedures, including transabdominal ultrasonography, helical CT with plain scan and enhanced scans, and magnetic resonance imaging (MRI) with magnetic resonance cholangiopancreatography and ERCP to confirm the diagnosis. All available imaging data including diagnosis reports and images were retrospectively reviewed by 2 radiologists. A consensus was reached with the main findings or signs recorded. Surgical and pathologic reports were also reviewed and correlated with the major findings or signs on comprehensive imaging. All the patients were discharged after sufficient recovery. This study was approved by the Institutional Review Board of the First Affiliated Hospital, Xi’an Jiaotong University, Xi’an, China.

### Follow-Up

During the first 6 months postoperatively, the patients were reexamined every 1–2 months. After that the patients were reexamined every 3–6 months. At each follow-up visit, clinical, laboratory, and radiological (abdominal CT scan and chest X-ray) data were collected. All 5 patients were followed-up until the end of 2012.

## RESULTS

### Patients’ Characteristics

The patients included 4 men and 1 woman between the age of 47 and 72 years. The characteristics of the patients are summarized in Table 1. Data from these patients were collected preoperatively, and notes from the referring hospital were reviewed whenever possible.

**TABLE 1:**
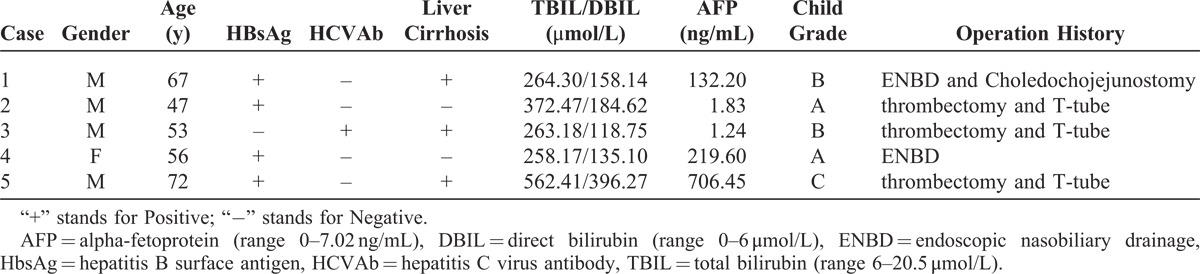
Characteristics of the Patients of HCC With BDTT at the Initial Diagnosis

### Classification of HCC With BDTT

HCC with BDTT was classified according to the classification proposed by Ueda et al,^11^ on the bases of the location of BDTT. HCC with BDTT was classified as type 1 (BDTT involving the second-order intrahepatic duct), type 2 (BDTT involving the first-order intrahepatic duct), type 3 (BDTT involving the hepatic confluence), and type 4 (dislodged BDTT within the common hepatic duct). The 5 cases were classified as shown in Table 2. According to Ueda et al classification,^11^ 4 cases belong to dislodged BDTT within the common hepatic duct or common bile duct and 1 case was BDTT involving the hepatic confluence.

**TABLE 2:**
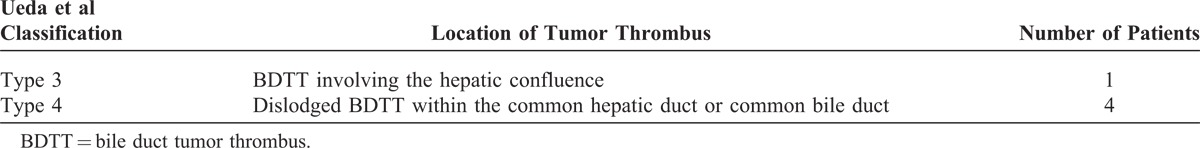
Classification of HCC With BDTT According to the Location of Thrombus

### Clinical Findings

Before operation, the average level of serum total bilirubin (TBIL) was 344.09 µmol/L (range 258.17–562.41 µmol/L) and direct bilirubin (DBIL) was 198.58 µmol/L (range 118.75–396.27 µmol/L). The concentration of alpha-fetoprotein (AFP) was 1.24–706.45 ng/mL, and was positive (>7.02 ng/mL) in 3 patients. Abnormal recurrent cholangitis was the predominant symptom in the patients and presented as an initial symptom in all 5 patients. The clinical presentations of the patients included right upper quadrant abdominal pain or upper abdominal pain, fever, jaundice, and elevated alkaline phosphatase level. BDTT could be seen in 2 patients by T-tube cholangiography. The other main clinical symptoms were fatigue, abdominal distension, loss of appetite, and loss of weight. Hepatitis B surface antigen was positive in 4 patients. Hepatitis C antibody was positive in 1 patient. The duration time of hepatitis B and C infection was more than 20 and 13 years, respectively, separately from the first time confirmed. Liver cirrhosis was found in 3 patients.

### Treatment Strategies

**FIGURE 1:**
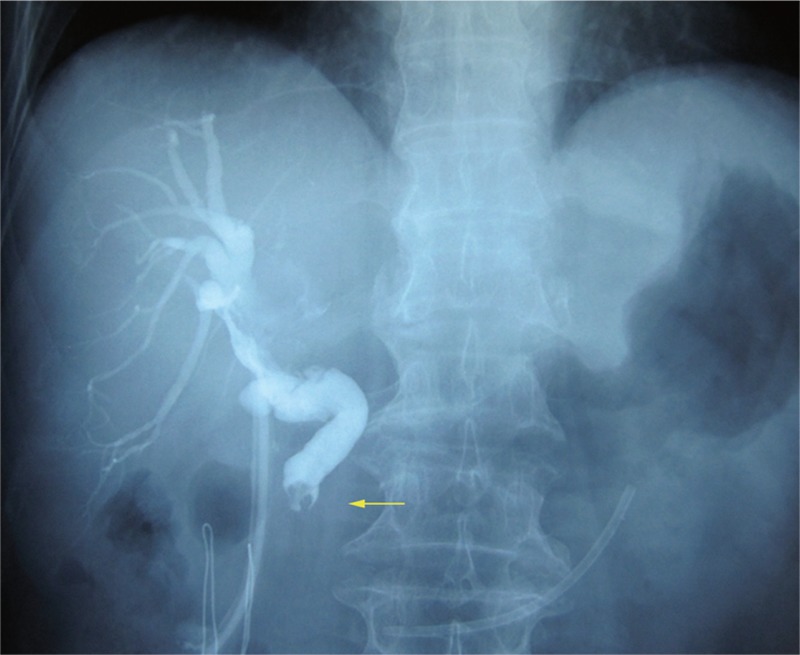
T-Tube cholangiography showing bile duct tumor thrombus (yellow arrow).

All five patients received emergency treatment in the local hospital when they were misdiagnosed as choledocholithiasis and cholangitis. Three patients received choledocholithotomy and T-tube drainage therapy and 2 of them were confirmed as BDTT pathologically after the operation. BDTT was detected again by T-Tube cholangiography after the patient was admitted to our hospital (Figure 1). These 2 patients underwent hepatectomy with thrombectomy and T-tube drainage after 14 and 20 days, respectively. The other patient was admitted to our hospital 4 months after the first treatment and only received percutaneous transhepatic biliary drainage (PTBD) therapy because of the portal vein tumor thrombus and intrahepatic metastasis. Two of 5 patients received emergency ENBD drainage, and 1 case was confirmed as BDTT after thrombus extraction during ERCP and then transferred to our hospital in 7 days. This patient underwent segments VII and VIII bisegmentectomy with thrombectomy and T-tube drainage. The last patient received plastic stent placement after ENBD drainage, and further received choledocholithotomy and choledochojejunostomy in the local hospital. This patient underwent left hemihepatectomy and hepatojejunostomy 92 days after the first treatment. The determination of resectability was based on tumor characteristics, remnant liver volume, liver function, and general status of the patients (Table 3).

**TABLE 3:**
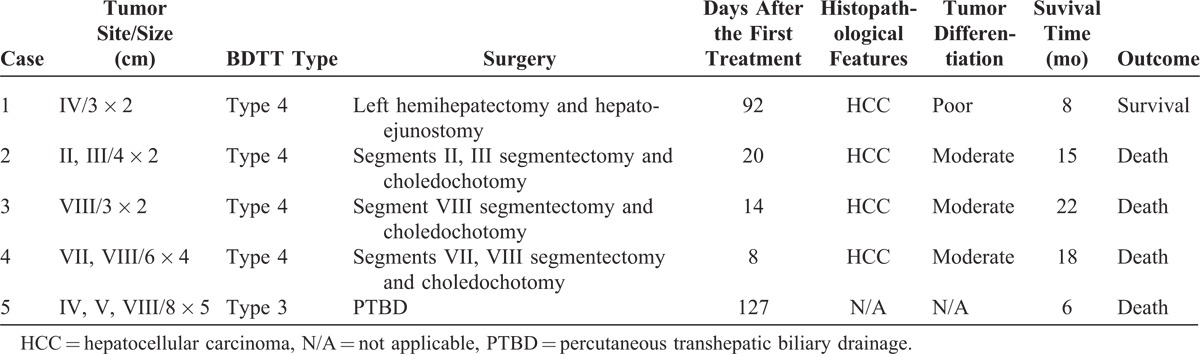
Summary of Clinical Characteristics During the Operation and Survival After Treatment of HCC With BDTT

### Outcome

Four patients underwent hepatectomy and BDTT removal. The extent of resection is shown in Table 3. One patient received PTBD therapy because of the portal vein tumor thrombus and intrahepatic metastasis. Surgical complications occurred in 1 patient, including pleural effusion and subphrenic abscess, that was successfully managed with conservative treatment. The patients were followed for 6–22 months. Four patients died of tumor recurrence and metastasis or hepatic failure with a mean survival time of 15 months, despite 3 of these patients received transhepatic arterial chemotherapy and embolization (TACE) or radiofrequency ablation therapy.

## DISCUSSION

HCC with BDTT has been reported to be rare and accounts for only 1.2%–9.0% of HCC.^12^ It is generally believed that the invasion of HCC into the biliary tree ultimately leads to the formation of BDTT. However, recent studies revealed that primary tumor might be small, even undetectable, and there was no histopathologic evidence of direct tumor invasion into bile duct wall in some patients.^13,14^ When the intrahepatic tumor is very small and cannot be detected by imaging examination, and recurrent cholangitis was the predominant symptom of the patients, it is difficult to distinguish HCC with BDTT from choledocholithiasis and cholangitis. In this study, 5 cases were misdiagnosed as choledocholithiasis and cholangitis in the local hospital because of cholangitis as the predominant symptom without intrahepatic tumor detection. Identification of this particular type of HCC is clinically important, because the presence of BDTT does not render HCC unresectable. After appropriate preoperative management, hepatectomy with thrombectomy appears to be effective for HCC patients with BDTT.

The mechanism of BDTT in HCC patients is not well understood. Previous reports demonstrated that HCC invades into the cystic duct to cause tumor thrombus shedding and biliary tract hemorrhage, which further lead to obstructive jaundice.^15^ The reasons why HCC is present as BDTT without detectable primary intrahepatic tumor are as follows; the tumor may originate from cancerization of ectopic hepatocytes in the bile duct wall, or the primary tumor is just too small to be identified, or the tumor located at the origin of or close to the intrahepatic duct grows intraluminally and stretchs inferiorly.^16^ With regard to the pathogenesis of BDTT, its formation is mainly through the following mechanisms^13,14,17^: HCC cells directly invades the bile duct and tumor tissues fill the bile duct; tumor tissues rupture and a fragment of tumor tissue separate from the primary lesion, migrate to different sites of the extrahepatic bile ducts, and result in obstruction jaundice and cholangitis; and hemorrhage from the tumor invasion may partially or completely fill the distal bile duct with tumor-containing blood clots. Generally, BDTT was not adherent to the bile duct wall so it could be removed easily. BDTT rarely invade the walls of the large bile ducts around the hepatic hilus. Therefore, liver resection of the involved hepatic segments with thrombectomy through a choledochotomy is a rational technique for curative resection.^12^ More recently, efforts on stem cell biology may shed light on the pathogenesis of BDTT. Accumulating evidences indicate that HCC with BDTT, especially with small or undetectable primary lesion and/or no histopathologic evidence for bile duct invasion, might arise from liver stem/progenitor cells residing in the canals of Hering and, possibly, some primary lesions are formed first within the intrahepatic biliary tree.^18^

Early and accurate diagnosis of HCC with BDTT is very important. In our cases, HCC patients with BDTT were misdiagnosed as choledocholithiasis because cholangitis was the predominant initial symptom. The primary suspect was a bile duct stone and the patients received choledochotomy or ENBD therapy at the initial admission in the local hospital. However, neither a bile duct stone nor a gallbladder stone had been shown in the past history. In this study, all the 5 patients were positive for the markers of chronic viral hepatitis. A relatively high percentage of cirrhosis was observed on preoperative images and during surgery in our patients. Although parenchymal mass may not be detectable on cross-sectional images in the BDTT patients, other signs were helpful in the diagnosis of HCC with BDTT, such as abnormal recurrent cholangitis, patients in the high-risk population with history of liver cirrhosis, hepatitis B surface antigen, or hepatitis C virus antibody positive. Patients with these features should be considered the potential diagnosis of HCC with BDTT and concentration of AFP levels should be detected for differential diagnosis of HCC. Intraductal ultrasonography (IDUS) is very valuable for diagnosing this disease. Sasaki et al^19^ reported that IDUS can distinguish between BDTT caused by HCC and bile duct stone according filling defect with or without acoustic shadow. However, IDUS has not been widely accepted because it is invasive and requires special equipments and specific expertise. The characteristics of early enhancement pattern on dual-phase contrast enhanced CT or dynamic contrast enhanced MRI are important to diagnosed BDTT.^20^ It was reported that color Doppler sonography can also effectively detect tumor vascularity of BDTT.^21^ Furthermore, if BDTT is suspected, it is still important to look for more sensitive techniques such as histopathological examination. Moreover, intraoperative ultrasonography (IOUS) should be performed to find the potential intrahepatic tumor or to determine the resection level, especially in patients with cirrhosis.^3^

The ideal therapy for HCC with BDTT is to remove the primary tumor and the BDTT surgically. Surgical methods include lobectomy of the liver, hepatectomy with removal of BDTT, and thrombectomy through choledochotomy followed by T tube drainage.^16,22^ As BDTT is not tightly adhesive to the bile duct wall, it is not difficult to remove during exploration of the biliary tract. However, although surgical treatment can achieve good results, most patients have missed the best time for surgery because of misdiagnose at the time of disease onset. Removal of BDTT via choledochotomy and resection of intrahepatic tumor were considered in patients with adequate hepatic function.^10^ Bile duct drainage, such as ENBD, plastic stent placement, PTBD, or PTBD plus stent placement, should be practiced to relieve jaundice in patients with extensive intrahepatic metastasis, multiple recurrent lesions or inadequate hepatic function. After bile duct drainage is performed, TACE may be performed to inhibit the vascularization of the tumor and the BDTT and thereby control tumor growth or even prevent fatal bile duct bleeding caused by the BDTT. HCC with BDTT may be accompanied by portal vein tumor thrombus simultaneously, as both the portal vein and bile duct are surrounded by the same glisson sheath.^14^ Portal vein tumor thrombus indicate systemic metastasis, which lead topoor prognosis, while the HCC that only combined BDTT could get good outcomes after treatment.

HCC patients with BDTT have a poorer prognosis than other HCC patients, probably because the following reasons:^10,12,22^ (a) many patients do not receive effective treatment at the best time for surgery because of misdiagnose at the initial admission to the local hospital. (b) HCC patients with BDTT is often accompanied by liver cirrhosis and obstructive jaundice, it is difficult to assess hepatic functional reserve before determining treatment. (c) In HCC patients with BDTT, shorter survival time may be associated with portal vein invasion, portal vein tumor thrombus and intrahepatic tumor recurrence.

In summary, early and accurate diagnosis of HCC with BDTT is very important. When patients have history of abnormal recurrent cholangitis, HCC with BDTT should be highly suspected. IDUS, IOUS and histopathological examination are very valuable for diagnosing this disease. The prognosis of HCC patients with BDTT is dismal. Identification of this type of patients is clinically important, because surgical treatment may be beneficial.
